# A Cytosolic Chaperone Complexes with Dynamic Membrane J-Proteins and Mobilizes a Nonenveloped Virus out of the Endoplasmic Reticulum

**DOI:** 10.1371/journal.ppat.1004007

**Published:** 2014-03-27

**Authors:** Christopher Paul Walczak, Madhu Sudhan Ravindran, Takamasa Inoue, Billy Tsai

**Affiliations:** 1 Department of Cell and Developmental Biology, University of Michigan Medical School, Ann Arbor, Michigan, United States of America; 2 Cellular and Molecular Biology Graduate Program, Ann Arbor, Michigan, United States of America; National Cancer Institute, United States of America

## Abstract

Nonenveloped viruses undergo conformational changes that enable them to bind to, disrupt, and penetrate a biological membrane leading to successful infection. We assessed whether cytosolic factors play any role in the endoplasmic reticulum (ER) membrane penetration of the nonenveloped SV40. We find the cytosolic SGTA-Hsc70 complex interacts with the ER transmembrane J-proteins DnaJB14 (B14) and DnaJB12 (B12), two cellular factors previously implicated in SV40 infection. SGTA binds directly to SV40 and completes ER membrane penetration. During ER-to-cytosol transport of SV40, SGTA disengages from B14 and B12. Concomitant with this, SV40 triggers B14 and B12 to reorganize into discrete foci within the ER membrane. B14 must retain its ability to form foci and interact with SGTA-Hsc70 to promote SV40 infection. Our results identify a novel role for a cytosolic chaperone in the membrane penetration of a nonenveloped virus and raise the possibility that the SV40-induced foci represent cytosol entry sites.

## Author Summary

The nonenveloped simian virus 40 (SV40) is a model member of the *Polyomaviridae* family of viruses containing several related species that cause diseases in immunocompromised individuals. As with other nonenveloped viruses, the membrane penetration step during SV40 entry is mechanistically obscure. Productive SV40 infection requires trafficking of the viral particle to the endoplasmic reticulum (ER) from where it penetrates the ER membrane to reach the cytosol; further transport of the virus into the nucleus causes infection. How SV40 crosses the ER membrane is an enigmatic step. Here, we identify a cytosolic chaperone protein that physically engages SV40 and facilitates virus ER-to-cytosol transport. This factor called SGTA is hijacked specifically at the site of membrane penetration due to its recruitment by ER membrane proteins B14 and B12 previously implicated in supporting virus infection. Additionally, we observe that B14 and B12 reorganize during SV40 entry into discrete foci on the ER membrane. These virus-induced structures likely represent exit sites for the viral particles and could serve to transiently recruit high concentrations of SGTA to complete membrane penetration. Our data reveal that a cytosolic chaperone can play a direct role in membrane penetration of a nonenveloped virus.

## Introduction

Nonenveloped viruses must penetrate a biological membrane to infect cells. As they lack a surrounding lipid bilayer, membrane penetration by nonenveloped viruses must be fundamentally different from enveloped viruses, which normally gain access to the host cell by membrane fusion. Although the precise membrane transport mechanism for nonenveloped viruses is not entirely clear, a general principle is emerging. These viruses enter host cells by endocytic internalization in order to arrive at a precise cellular environment necessary for productive infection [1]. Upon reaching this proper environment, important conformational changes are induced by specific cellular triggers including low pH, proteases, or chaperone activities [2]. These conformational changes in turn generate a hydrophobic viral particle or cause the release of a lytic peptide hidden in the intact virus. Engagement of the hydrophobic particle or lytic peptide with the limiting membrane disrupts the membrane integrity and initiates membrane penetration. For example, the nonenveloped reovirus, parvovirus, and adenovirus become internalized and traffic to endosomes where the low pH or proteases trigger viral conformational changes that allow them to penetrate the endosomal membrane [3]–[6]. In these cases, membrane penetration is thought to involve virus-induced pore formation or disruption of overall membrane integrity. Currently, absent in this model is a role for any cytosolic factors directly influencing membrane penetration.

Polyomaviruses are unique among nonenveloped viruses in that they traffic beyond the endosomal system to reach the endoplasmic reticulum (ER) for membrane penetration [7]–[13]. This virus family consists of a growing list of important human polyomaviruses known to cause devastating diseases in immunocompromised individuals [14], [15]. Simian virus 40 (SV40) has traditionally served as an excellent model member of this family; it has genetic and structural similarity to human polyomaviruses, yet is easy to propagate and study in cells. To cause infection, SV40 engages the ganglioside receptor GM1 at the cell surface to initiate internalization [16], [17]. Caveolae-dependent endocytosis brings SV40 particles attached to lipid rafts into the cell where they travel through endosomes before being sorted to the ER [7], [12]. Once inside the ER lumen, SV40 is faced with the task of penetrating the ER membrane to reach the cytosol prior to nuclear import [18], [19]. In the nucleus, transcription and replication of the viral genome are initiated, leading to lytic infection or cellular transformation.

The ER provides an ideal environment for inducing important conformational changes to the structure of SV40. The outer surface of each viral particle contains 360 copies of the major coat protein VP1 arranged as 72 pentamers. A single hydrophobic minor coat protein VP2 or VP3 resides beneath each VP1 pentamer [20]. VP1 molecules are stabilized by interpentameric disulfide bonds, with bound calcium ions and hydrophobic interactions providing additional capsid support [21], [22]. Protein disulfide isomerase (PDI)-family members appear to be broadly important during entry of polyomaviruses for either their ability to disrupt viral disulfide bonds by using their redox/isomerase activities, or to impart conformational changes by using their chaperone functions [10], [23]–[26]. PDI proteins exert these activities on SV40, likely in concert with other ER factors, resulting in VP2 exposure [9], [24], [27], [28]. Due to its hydrophobic N-terminus, exposure of VP2 renders the virus itself hydrophobic. As a result, the virus binds and integrates into the ER membrane to initiate membrane penetration [27], [29], [30].

In the next stage of the membrane penetration process, a critical Glu residue in VP2's N-terminus embedded in the ER membrane is hypothesized to act as a charged irregularity in the ER membrane and recruits cellular factors involved in ER-associated degradation (ERAD) [29]. The ERAD pathway utilizes large multi-protein complexes to eliminate misfolded or incorrectly assembled ER proteins by facilitating their retro-translocation into the cytosol for degradation by the ubiquitin-proteasome system [31], [32]. SV40 and other polyomaviruses co-opt several ERAD membrane components including the J-proteins DnaJB14 (B14) and DnaJB12 (B12) to reach the cytosol and infect cells [10], [11], [23], [29], [33]–[35]. B14 and B12 both span the ER membrane once and display their functional J domain in the cytosol [36]–[38]. By virtue of this domain, J-proteins stimulate the ATPase activity of Hsp70 chaperones to promote substrate-Hsp70 interaction [39]. However, the precise mechanism by which these membrane components facilitate ER-to-cytosol transport of a large viral particle [40] is not clear. Whether any cytosolic components provide the driving force to extract the hydrophobic ER membrane-embedded SV40 into the cytosol is also unknown. In this context, the cytosolic ATPase p97 (also called VCP) involved in the mobilization of ERAD substrates into the cytosol [41] was shown to be dispensable for SV40 infection [29]. Additionally, while chemical inhibition of the cytosolic proteasome perturbs infection of SV40 and other polyomaviruses [11], [23], [34], [40], this effect may be indirect [29]. Thus, the potential roles of cytosolic factors that directly promote membrane penetration of polyomaviruses, as well as other nonenveloped viruses, remain enigmatic.

Here, we demonstrate that the cytosolic chaperone SGTA (small glutamine-rich tetratricopeptide repeat-containing protein α) is critical for transport of SV40 from the ER membrane into the cytosol. SGTA associates with a chaperone complex containing B14 and B12 (B14-B12) at the ER membrane and is therefore positioned to act at the site of membrane penetration. Using a combination of cell-based and biochemical assays, we found that SGTA binds directly to SV40 and promotes its ER membrane penetration and infection. During membrane penetration, SGTA is released from the B14-B12 complex, suggesting SV40 alters localization or structural characteristics of these factors. Consistent with this idea, we found that SV40 causes the B14-B12 complex to reorganize into discrete foci in the ER membrane. As formation of these foci coincides temporally with SV40's cytosolic arrival, they may represent ER exit sites where SGTA engages the virus to complete membrane penetration.

## Results

### B14 Forms a High Molecular Weight Complex with B12

**Figure 1:**
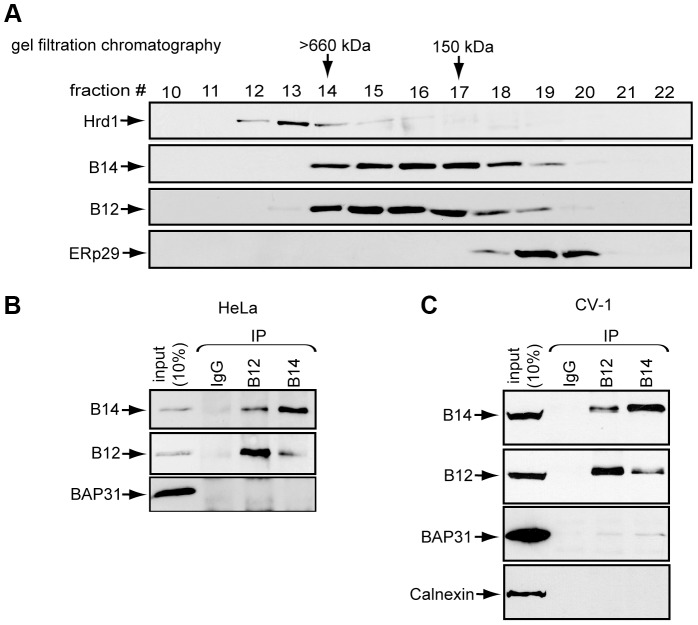
B14 complexes with B12. (A) Lysates prepared from HeLa cells were subjected to gel filtration chromatography. Collected fractions were analyzed by immunoblot with the indicated antibodies. (B) B14 and B12 interact. HeLa cell lysates were used for immunoprecipitation with control IgG or antibodies against endogenous B12 or B14. (C) As in (B) except CV-1 cell lysate was used.

To clarify how B14 and B12 promote SV40 ER membrane penetration [33], we first characterized their biochemical properties. Gel-filtration analysis of detergent-solubilized cell extracts followed by subsequent immunoblotting of the individual fractions demonstrated that a substantial pool of B14 eluted in high molecular weight fractions (>150 kDa), suggesting B14 is part of a complex larger than its monomeric size of 42 kDa (Figure 1A). Probing for Hrd1 and ERp29 confirmed the fractionation of large and small complexes, respectively. B12 co-fractionated identically with B14, raising the possibility that these proteins interact with each other. Co-immunoprecipitation experiments revealed that endogenous B14 and B12 bind to each other with high efficiency in both HeLa and CV-1 cells (Figure 1B and 1C). These data demonstrate that these J-proteins interact with each other and are likely part of a stable membrane complex.

### The Cytosolic Chaperone SGTA Binds to the B14-B12 Complex in a Hsc70-Dependent Manner

**Figure 2:**
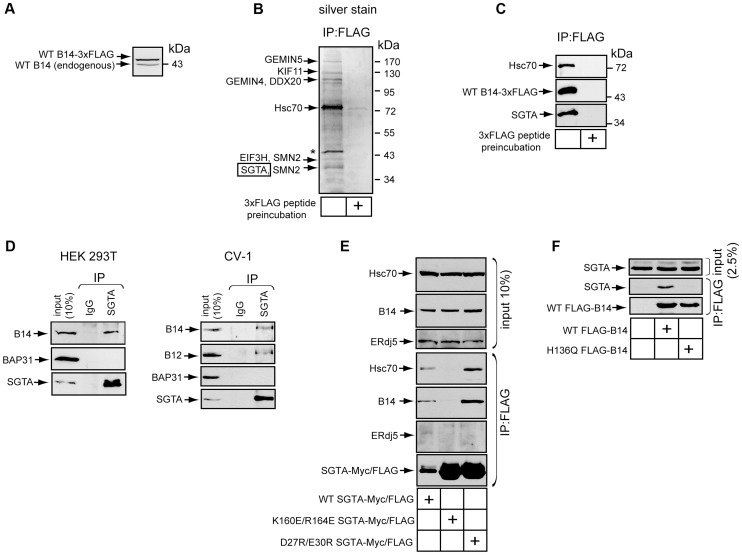
The cytosolic chaperone SGTA binds to the B14-B12 complex in a Hsc70-dependent manner. (A) B14-3xFLAG is expressed at a level near endogenous B14. Whole cell lysates derived from Flp-In 293 T-REx (B14-3xFLAG) cells were analyzed by SDS-PAGE and immunoblotted with an antibody against B14. Relative molecular weight markers in kDa are shown on the right. (B) SGTA identified by MS as a binding partner of B14. Immunopurified B14-3xFLAG complexes from lysate in (A) was eluted by 3xFLAG peptide and subjected to SDS-PAGE and silver staining. A control experiment was performed in which lysate was incubated with anti-FLAG agarose beads pre-incubated with 3xFLAG peptide. Indicated proteins were identified after band excision using MS. * indicates a protein migrating at the size of B14-3xFLAG. (C) As in (B) except immunoblotting was performed with the indicated antibodies. (D) Endogenous interaction between SGTA and B14-B12. Lysates derived from 293T (left) or CV-1 cells (right) were used for immunoprecipitation with the indicated antibodies. (E) SGTA's interaction with B14 requires coupling to Hsc70. CV-1 cells were transfected with plasmids expressing FLAG-tagged WT or SGTA mutants. Immunoprecipitation was performed from the cell lysates using anti-FLAG agarose beads with subsequent immunoblot analysis. (F) As in (E) except cells were transfected with plasmids encoding WT or mutant B14.

To identify other components of the B14 complex, we used an unbiased strategy of immunoprecipitation followed by mass spectrometry (MS). We constructed 293T cells that allowed for tetracycline-inducible expression of B14-3xFLAG. To minimize overexpression artifacts, B14-3xFLAG expression was maintained at a level similar to endogenous B14 by providing a low concentration of tetracycline (Figure 2A). Under this condition, cells were lysed with a low concentration of digitonin to maintain protein-protein interactions during immunoprecipitation with anti-FLAG conjugated agarose beads. To control for nonspecific binding to the agarose beads, an identical lysate was incubated with anti-FLAG conjugated agarose beads pre-blocked with 3xFLAG peptide. After washing and elution by addition of 3xFLAG peptide, the eluted material was concentrated and subjected to SDS-PAGE followed by silver staining (Figure 2B). Co-immunoprecipitated proteins reproducibly observed by silver staining were excised and analyzed by MS. Not surprisingly, a band migrating at approximately 72 kDa was identified as Hsc70, a common interacting partner of J-proteins. Interestingly, another band corresponding to approximately 38 kDa was identified as the cytosolic chaperone SGTA. We focused our study on this protein as it was recently implicated in the ERAD pathway [42]. While several components of the SMN complex involved in spliceosomal snRNP assembly and pre-mRNA processing were also identified during MS analyses, they remain to be tested as authentic B14 binding partners. Eluted samples prepared as in Figure 2B were immunoblotted with specific antibodies to confirm the presence of SGTA, Hsc70, and B14-3xFLAG (Figure 2C). Importantly, when endogenous SGTA was immunoprecipitated from standard 293T or CV-1 cells, endogenous B14 but not the abundant membrane protein BAP31 was detected in the precipitate (Figure 2D, left and right panels). As B12 complexes with B14 (Figure 1), we asked whether endogenous B12 also binds SGTA. Indeed, B12 also co-precipitated with SGTA. (Figure 2D, right panels).

We next investigated whether the interaction between SGTA and B14-B12 was direct or mediated by a previously reported SGTA binding partner. SGTA has been observed to interact with Hsc70 as a co-chaperone [43], [44]. Additionally, a more recent study demonstrated that SGTA binds to the cytosolic Bag6 (BAT3, scythe)-Ubl4a-Trc35 complex via Ubl4a [42]. We therefore tested whether previously characterized SGTA mutants defective in their ability to bind to either Hsc70 (K160E/R164E) or Ubl4a (D27R/E30R) [42] could interact with B14. CV-1 cells were transfected to express FLAG-tagged WT or a mutant form of SGTA. Lysates derived from these cells were immunoprecipitated with anti-FLAG conjugated agarose beads. Endogenous B14 was observed to precipitate with WT-SGTA and the Ubl4a-binding defective mutant (D27R/E30R) (Figure 2E). By contrast, the Hsc70-binding defective mutant (K160E/R164E) was unable to interact with B14, despite substantially more mutant in the precipitation when compared to WT SGTA (Figure 2E). This interaction was specific to B14's cytosolic J domain as ERdj5, a lumenal J-protein, did not precipitate with any form of SGTA (Figure 2E). We performed the converse analysis by transfecting FLAG-tagged WT B14 or a mutant B14 defective in coupling to Hsc70 (H136Q) [37]. WT B14 but not H136Q B14 precipitated endogenous SGTA (Figure 2F). Together these data demonstrate SGTA interacts with the B14-B12 complex in a Hsc70-dependent manner.

### SGTA Knockdown Perturbs SV40 and BKPyV Infection

**Figure 3:**
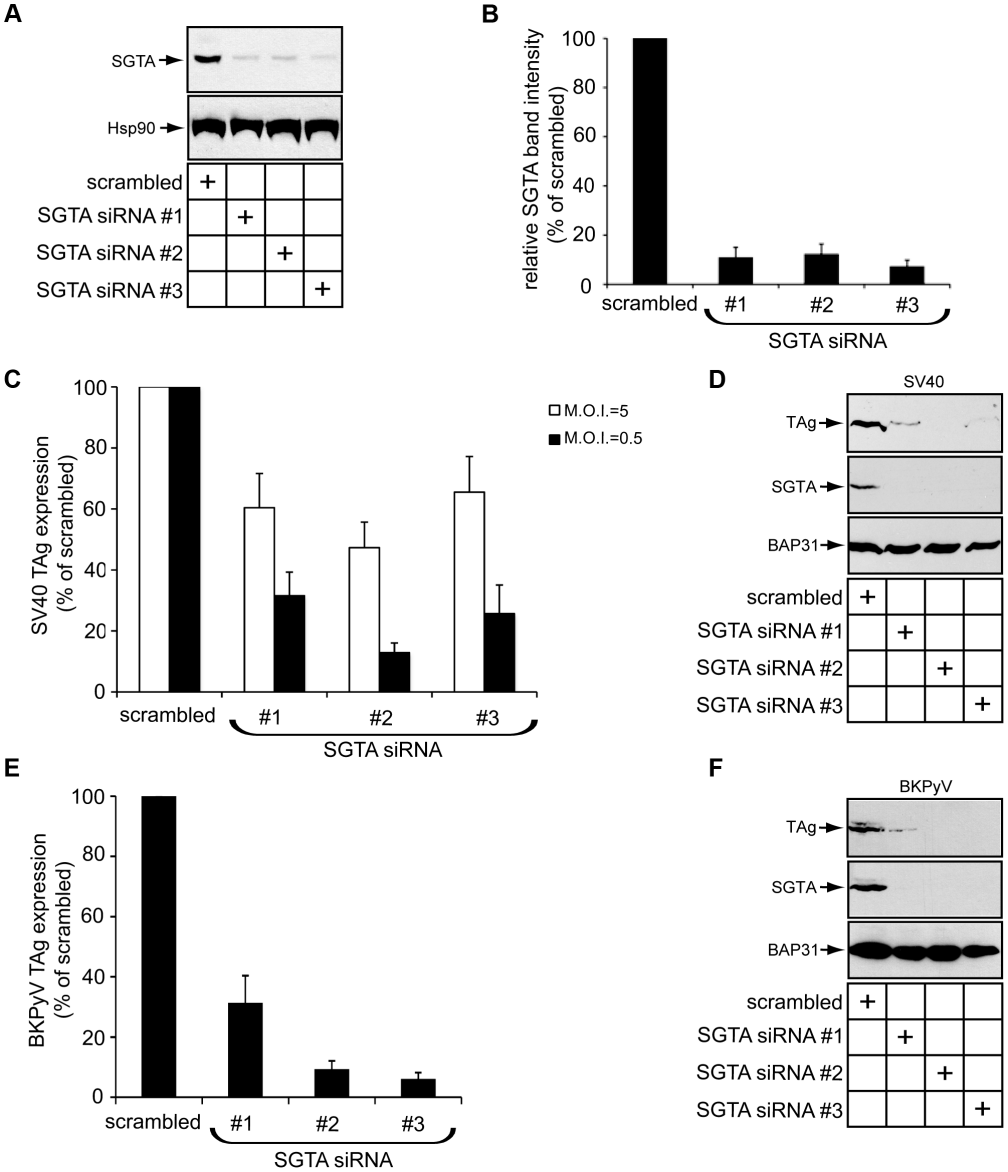
SGTA knockdown perturbs SV40 and BKPyV infection. (A) CV-1 cells were transfected with scrambled or siRNAs targeting SGTA for 36 h before being harvested and analyzed by immunoblotting with the indicated antibodies. (B) Quantification of the relative SGTA band intensities from (A) were determined using ImageJ (NIH). Data are normalized to scrambled siRNA. Values represent the mean ± SD of three independent experiments. A statistically significant (*p*<0.05) difference was observed with all SGTA knockdown conditions compared to scrambled. (C) SGTA knockdown blocks SV40 infection. CV-1 cells transfected for 24 h were infected with SV40 for 20–24 h, fixed, and stained for TAg. Infection was scored using immunofluorescence microscopy (counting ≥500 cells for each condition). With scrambled transfected cells, infection at a M.O.I. ≈5 or 0.5 resulted in 30–50% or 5–10% cells positive for TAg, respectively. A statistically significant (*p*<0.05) difference was observed with all SGTA knockdown conditions compared to scrambled. (D) CV-1 cells transfected and infected with SV40 (M.O.I ≈0.5) as in (C). Cells were harvested and lysed 40 h.p.i. followed by immunoblotting with the indicated antibodies. (E) SGTA knockdown blocks BKPyV infection. As in (C) except BKPyV was used (M.O.I. ≈0.5) and cells were stained at about 40 h.p.i. (F) As in (D) except BKPyV (M.O.I ≈0.5) was used.

B14 and B12 are two key ER membrane components crucial for SV40 and human BK polyomavirus (BKPyV) infection [33]. Since our findings revealed SGTA is a binding partner of the B14-B12 complex, we first asked whether SGTA is also important for SV40 infection. Expression of the virally-encoded large T antigen protein (TAg) in the host nucleus reflects successful viral infection. We monitored infection in this way by scoring cells for the presence or absence of TAg using immunofluorescence microscopy. Three distinct siRNA oligonucleotides effectively downregulated SGTA expression in CV-1 cells by over 80% (Figure 3A; quantified in 3B). Using a low M.O.I. (i.e. 0.5), silencing SGTA robustly inhibited SV40 infection by 70–80% when compared to the scrambled control (Figure 3C, black bars). When a higher M.O.I. (i.e. 5) was used, SGTA knockdown decreased infection by approximately 40–50% (Figure 3C, white bars). A striking decrease in TAg expression was also evident by immunoblot analysis of whole cell extracts derived from infected cells (Figure 3D). Similarly, these knockdown conditions markedly blocked the expression of BKPyV TAg as assessed by immunofluorescence and immunoblot analyses (Figure 3E and 3F). We conclude that SGTA plays an important role during SV40 and BKPyV infection.

SGTA is involved in multiple aspects of protein quality control [42], [45]. Recent evidence suggests one function of SGTA is to facilitate ERAD substrate loading on Bag6, a holdase that prevents substrate aggregation prior to proteasomal degradation [42], [46]. Based on these findings, we tested whether Bag6 was important for SV40 infection and found that Bag6 knockdown did not block expression of SV40 TAg (Figure S1), indicating that SGTA promotes SV40 infection independent of Bag6.

### SGTA Knockdown Blocks Viral ER-to-Cytosol Transport

**Figure 4:**
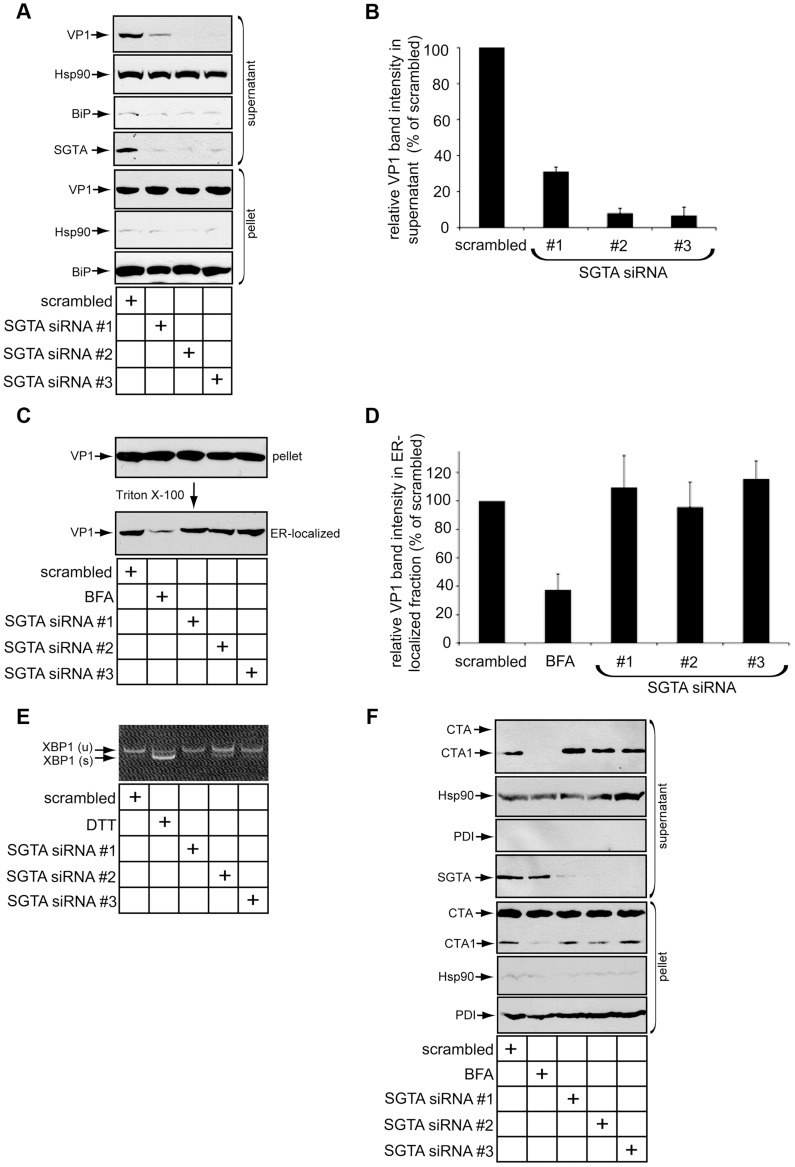
SGTA knockdown blocks viral ER-to-cytosol transport. (A) SGTA promotes ER-to-cytosol transport of SV40. CV-1 cells transfected for 24 h with the indicated siRNAs were incubated with SV40 at a MOI ≈5, harvested 12 h.p.i. and subjected to ER-to-cytosol transport assay (see experimental procedures). BiP and Hsp90 serve as markers for the ER and cytosol, respectively. (B) Relative VP1 band intensities in the supernatant fractions from (A) were determined using ImageJ. Data are normalized to scrambled siRNA. Values represent the mean ± SD of three independent experiments. A statistically significant (*p*<0.05) difference was observed with all SGTA knockdown conditions compared to scrambled. (C) Loss of SGTA does not block ER arrival of SV40. Pellet fraction produced from cells as in (A) were solubilized in a buffer containing 1% Triton X-100. After centrifugation, the soluble fractions were analyzed by VP1 immunoblotting. As a positive control, where indicated, cells were treated with BFA to block ER arrival. (D) Relative VP1 band intensities in Triton X-100 soluble (ER-localized) fractions from (C) were determined using ImageJ. Data are normalized to scrambled siRNA. Values represent the mean ± SD of three independent experiments. A statistically significant (*p*<0.05) difference was not observed with any SGTA knockdown conditions compared to scrambled. (E) XBP1 splicing does not occur with transient SGTA knockdown. RT-PCR analysis of the unspliced (u) and spliced (s) forms of XBP1 mRNA in cells transfected with scrambled or SGTA siRNAs. Where indicated, cells were treated with DTT as a positive control for ER stress induction. (F) CTA1 retro-translocation is not blocked by SGTA knockdown. CV-1 cells were transfected with the indicated siRNAs for 36–40 h before CT intoxication at 10 nM for 90 mins. Cells were harvested and fractionated as in (A). PDI and Hsp90 serve as markers for the ER and cytosol, respectively. A shorter exposure of CTA is provided for the pellet fraction to clearly display input toxin.

As SGTA is a cytosolic chaperone complexed with the ER membrane J-proteins B14 and B12, we asked whether this chaperone facilitates the arrival of SV40 into the cytosol from the ER. Our laboratory previously developed a cell-based assay to monitor SV40 ER-to-cytosol transport [40]. A similar assay has also been reported [29]. Briefly, cells infected with SV40 are harvested, selectively permeabilized with a low digitonin concentration, and centrifuged to generate a supernatant and a pellet fraction; the supernatant harbors cytosolic material while the pellet contains intracellular membranes including the ER. These fractions are subsequently analyzed by immunoblotting for fractionation markers and VP1. VP1 in the supernatant therefore represents SV40 that reached the cytosol, and VP1 in the pellet reflects virus within intracellular organelles. We applied this assay to cells transfected with scrambled or SGTA siRNAs. The cytosolic marker Hsp90 was predominantly detected in the supernatant whereas the ER lumenal marker BiP was found exclusively in the pellet (Figure 4A), confirming the integrity of the fractionation procedure. Strikingly, the VP1 level in the supernatant was significantly reduced in cells with SGTA downregulated when compared to the scrambled control (Figure 4A; quantified in 4B). We note that the severity of the ER-to-cytosol transport defect, while consistent with the robust block in infection when cells were incubated with a low M.O.I. (Figure 3B, black bar), appears to be greater than the perturbation in infection when a high M.O.I. was used (Figure 3B, white bars). This difference is likely attributed to the remaining low amounts of virus in the cytosol that are sufficient to reach the nucleus to express TAg [47].

We also assessed whether downregulating SGTA affects arrival of SV40 to the ER. Successful SV40 ER arrival can be monitored by examining the amount of Triton X-100 soluble VP1 present in the pellet (i.e. membrane fraction). We previously found that only viruses which arrive in the ER are released from Triton X-100 insoluble lipid rafts into the ER lumen whose content can be extracted by Triton X-100 [40]. As expected, when cells were infected in the presence of brefeldin A (BFA), a drug that blocks COPI-dependent retrograde transport from the plasma membrane to the ER, there was a robust block in ER arrival of SV40 (Figure 4C; quantified in 4D) [13]. However, no detectable loss of SV40 ER arrival was apparent when SGTA was downregulated (Figure 4C; quantified in 4D). Thus SGTA does not significantly regulate retrograde trafficking of SV40 to the ER. Given that a pool of SGTA resides on the cytosolic side of the ER membrane due to its interaction with B14 and B12, we propose this factor facilitates SV40 infection primarily by completing the membrane penetration step during entry.

We assessed whether SGTA knockdown causes massive ER stress, which may explain the observed disruption of SV40 transport across the ER membrane. Monitoring the splicing of XBP1 mRNA (which encodes a stress-responsive transcription factor) is a sensitive method for detecting ER stress induction. When SGTA was silenced, we detected modest splicing of this transcription factor mRNA (SGTA siRNA #2), in contrast to cells exposed to the chemical ER stress inducer dithiothreitol (DTT) (Figure 4E). This finding indicates that significant ER stress was not triggered by silencing SGTA. While this result varies slightly from a recent report in which 293T cells expressing SGTA shRNA exhibited a strong ER stress response [42], this difference may be due to our use of a transient knockdown system in CV-1 cells.

Cholera toxin (CT) is another pathogenic factor that becomes internalized and traffics to the ER. From the ER, the catalytic A1 subunit of CT (CTA1) retro-translocates to the cytosol to induce cytotoxicity. In fact, CTA1 retro-translocation requires some of the same ERAD machinery co-opted by SV40 [48]–[50]. Unlike SV40, we found that SGTA knockdown did not inhibit transport of CTA1 to the cytosol (Figure 4F), whereas BFA treatment potently blocked CTA1 retro-translocation (Figure 4F), as previously reported [50]. These data indicate that defective SV40 ER-to-cytosol transport and infection caused by SGTA silencing was not due to poor cell health or general ER dysfunction.

### SGTA and Hsc70 Dissociate from the B14-B12 Complex during SV40 Entry

**Figure 5:**
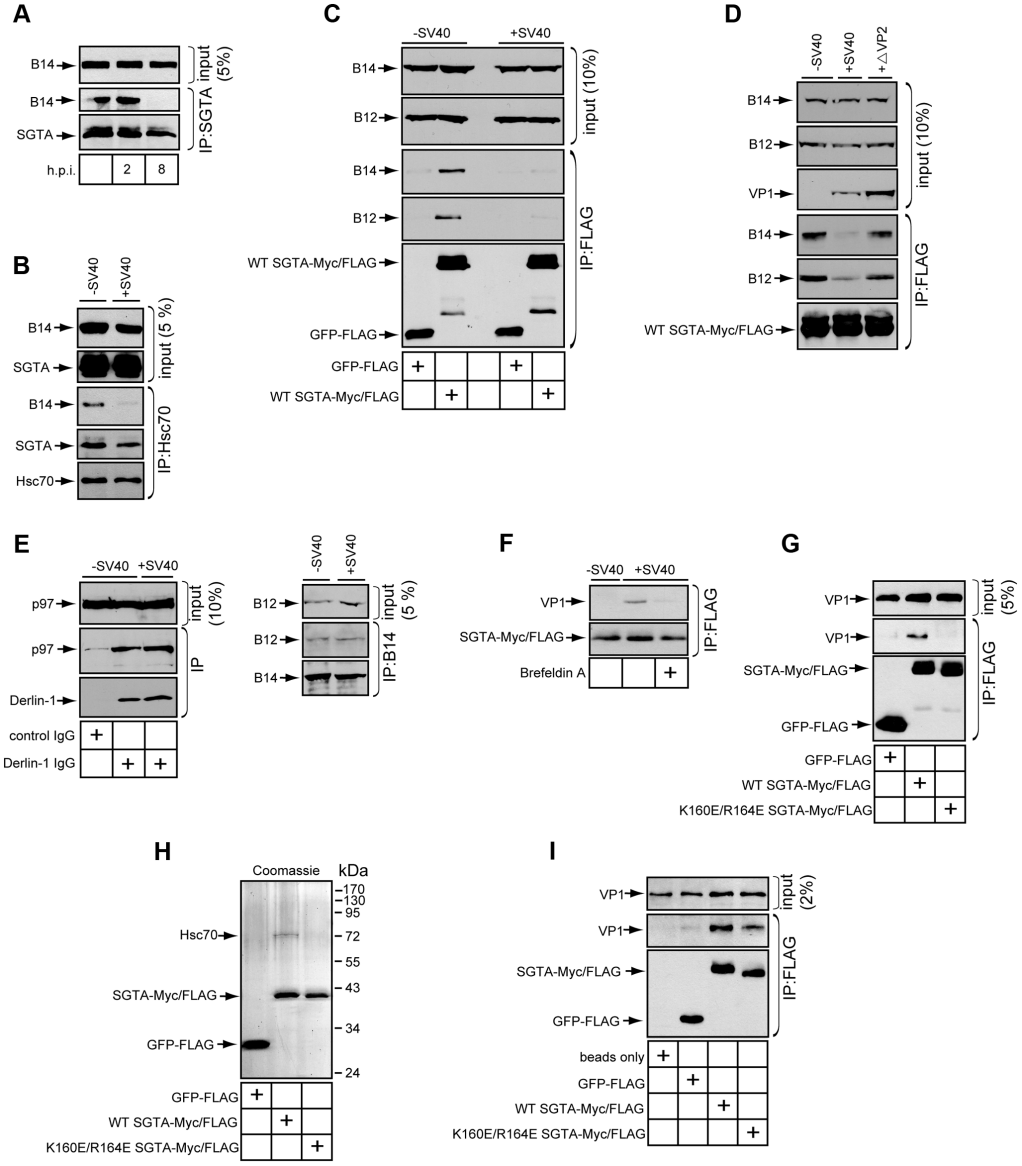
SGTA-Hsc70 dissociates from the B14-B12 complex and engages SV40 during entry. (A) Endogenous SGTA is released from B14 during entry. CV-1 cells were uninfected or infected with SV40 at a high MOI (≈100) for the indicated time. Cells were harvested, cross-linked, and lysed in buffer containing 1% Triton X-100 followed by immunoprecipitation with SGTA antibodies and immunoblotting. (B) Endogenous Hsc70 is released from B14 during entry. CV-1 cells were infected or uninfected for 12 h and immunoprecipitated as in (A) except Hsc70 antibodies were used. (C) Transfected SGTA is released from B14-B12 during entry. CV-1 cells transfected to express FLAG-tagged GFP or SGTA were infected with SV40 for 16 h. Immunoprecipitation was performed without cross-linking and the precipitated material analyzed by immunoblotting. (D) SV40 lacking VP2 fails to trigger dissociation of SGTA from B14-B12. As in (C) except cells were also infected with ΔVP2 SV40. (E) p97-Derlin-1 interaction is unaffected by SV40. Cells infected with virus as in (A) were lysed without cross-linking and immunoprecipitated with the indicated antibodies with subsequent immunoblotting of the precipitated material. (F) SGTA interacts with SV40 in cells during entry. CV-1 cells transfected to express FLAG-tagged WT SGTA were infected with SV40 at high MOI. Where indicated, cells were treated with BFA. Cells were cross-linked, lysed, and immunoprecipitated with anti-FLAG agarose beads. Precipitated proteins were analyzed by immunoblotting with the indicated antibodies. (G) SGTA requires localization to B14-B12 for interaction with SV40. As in (F) except cells were transfected to express either FLAG-tagged GFP, WT SGTA or mutant SGTA. (H) Coomassie staining of purified proteins. (I) Direct binding of SGTA and SV40 *in vitro*. Purified proteins were incubated with DTT/EGTA-treated SV40. Reactions were cross-linked, and the sampled precipitated using anti-FLAG agarose beads. Bound material was analyzed by immunoblotting.

If SGTA extracts SV40 from the ER membrane into the cytosol, we reasoned that SGTA itself might dissociate from the B14-B12 complex during virus transport. We first monitored SGTA-B14 interaction using co-immunoprecipitation assays during SV40 entry. Cells were uninfected or infected for 2 or 8 h, harvested, followed by cross-linking of the intact cells. Similar to previous experiments (Figure 2D), when endogenous SGTA was immunoprecipitated, a significant amount of B14 was detected (Figure 5A). This interaction remained intact at 2 h post infection (h.p.i.) (Figure 2D), a time point when SV40 particles are largely present in endosomes and have not yet reached the ER [7], [12]. By contrast, a stable SGTA-B14 interaction was completely lost at 8 h.p.i. (Figure 5A) when SV40 has reached the ER and initiated ER-to-cytosol transport [29], [40]. When Hsc70 was immunoprecipitated under similar conditions, a loss of stable B14 interaction was also detected (Figure 5B). However, the interaction between Hsc70 and SGTA was mostly preserved. These data indicate that during time points of entry where SV40 is undergoing membrane penetration, SGTA and Hsc70 are being released from the B14-B12 complex at the ER membrane.

This phenomenon held true in CV-1 cells transfected to express FLAG-tagged GFP or FLAG-tagged SGTA (Figure 5C); when cells uninfected or infected for 16 h were lysed and immunoprecipitated using anti-FLAG agarose beads, stable interactions between transfected SGTA and endogenous B14 as well as B12 remained disrupted at this later time point. While membrane penetration is observed to begin between 6 and 8 h.p.i., this process likely continues at later times due to the asynchronous nature of SV40 entry [7], [51].

Our time point analyses suggested that SV40 penetration across the ER membrane is required to release SGTA from the B14-B12 complex. To further test this hypothesis, we utilized a mutant SV40 lacking VP2 (i.e. ΔVP2 SV40). This mutant virus becomes internalized but fails to penetrate the ER membrane to reach the cytosol [29]. When compared to WT SV40, ΔVP2 SV40 did not disrupt the interaction of SGTA and B14-B12 (Figure 5D). Together, our findings indicate that the initiation of ER membrane penetration by SV40 is required for release of SGTA from the B14-B12 complex.

We assessed whether this dissociation event was general for ER bound cytosolic chaperones or specific to SGTA. The cytosolic p97 chaperone is well documented to link to ERAD complexes at the ER membrane and facilitate retro-translocation of ERAD substrates [41], [52], [53]. In cells infected with SV40, p97's interaction with the ERAD membrane component Derlin-1 was unchanged (Figure 5E). This result is consistent with the recent finding that p97 is not required for SV40 entry and infection [29]. SV40 infection also did not disrupt endogenous B14-B12 interaction (Figure 5E). We conclude SV40 specifically triggers SGTA to be released from the B14-B12 complex without globally disrupting the connection of cytosolic factors to the ER membrane.

### SGTA Associates with SV40 during Entry into Cells and *In Vitro*

An additional explanation to account for the release of SGTA from B14-B12 is that SGTA disengages from B14-B12 in order to engage SV40 as it becomes exposed in the cytosol. To test this possibility, CV-1 cells were transfected to express FLAG-tagged SGTA and infected for 8 h followed by cross-linking and immunoprecipitation with anti-FLAG agarose beads. Indeed, VP1 was detected in the immunoprecipitate of infected cells (Figure 5F). By contrast, VP1 was not detected in infected cells treated with BFA, which blocks ER arrival and subsequent transport to the cytosol (Figure 5F). Thus, SGTA binds to SV40 upon cytosolic arrival. We next investigated whether this SV40-SGTA interaction required the Hsc70-dependent localization of SGTA to the B14-B12 complex. When cells transfected with FLAG-tagged GFP, WT SGTA or the Hsc70-binding defective SGTA mutant (K160E/R164E) were infected and subjected to immunoprecipitation, VP1 was observed to co-precipitate only with WT SGTA and not GFP or K160E/R164E SGTA (Figure 5G). This result suggests that localization of SGTA to the B14-B12 complex via Hsc70 is required for SGTA to engage the virus during entry.

Hsc70 has been reported to associate with polyomaviruses *in vitro* and in cells during both entry and *de novo* capsid assembly [54], [55]. To assess whether SGTA binds to SV40 directly or requires Hsc70, we tested whether the SGTA-SV40 interaction could be recapitulated *in vitro* using purified components. FLAG-tagged GFP, WT SGTA and K160E/R164E SGTA were purified from transfected 293T cells. WT but not GFP or K160E/R164E SGTA copurified Hsc70 (Figure 5H). These purified components were incubated with purified SV40 pretreated with DTT and EGTA to partially mimic conformational changes that occur in the ER [23], [24]. When GFP or SGTA proteins were immunoprecipitated from these reactions, VP1 was detected only in reactions containing SGTA (Figure 5I). WT SGTA pulled down moderately more VP1 when compared to K160E/R164E SGTA, likely due to the presence of Hsc70 in the WT SGTA preparation. Nonetheless, the observation that purified K160E/R164E SGTA lacking copurified Hsc70 binds to VP1 demonstrates that SGTA can directly interact with SV40.

### SV40 Induces the B14-B12 Complex to Form Foci on the ER Membrane

**Figure 6:**
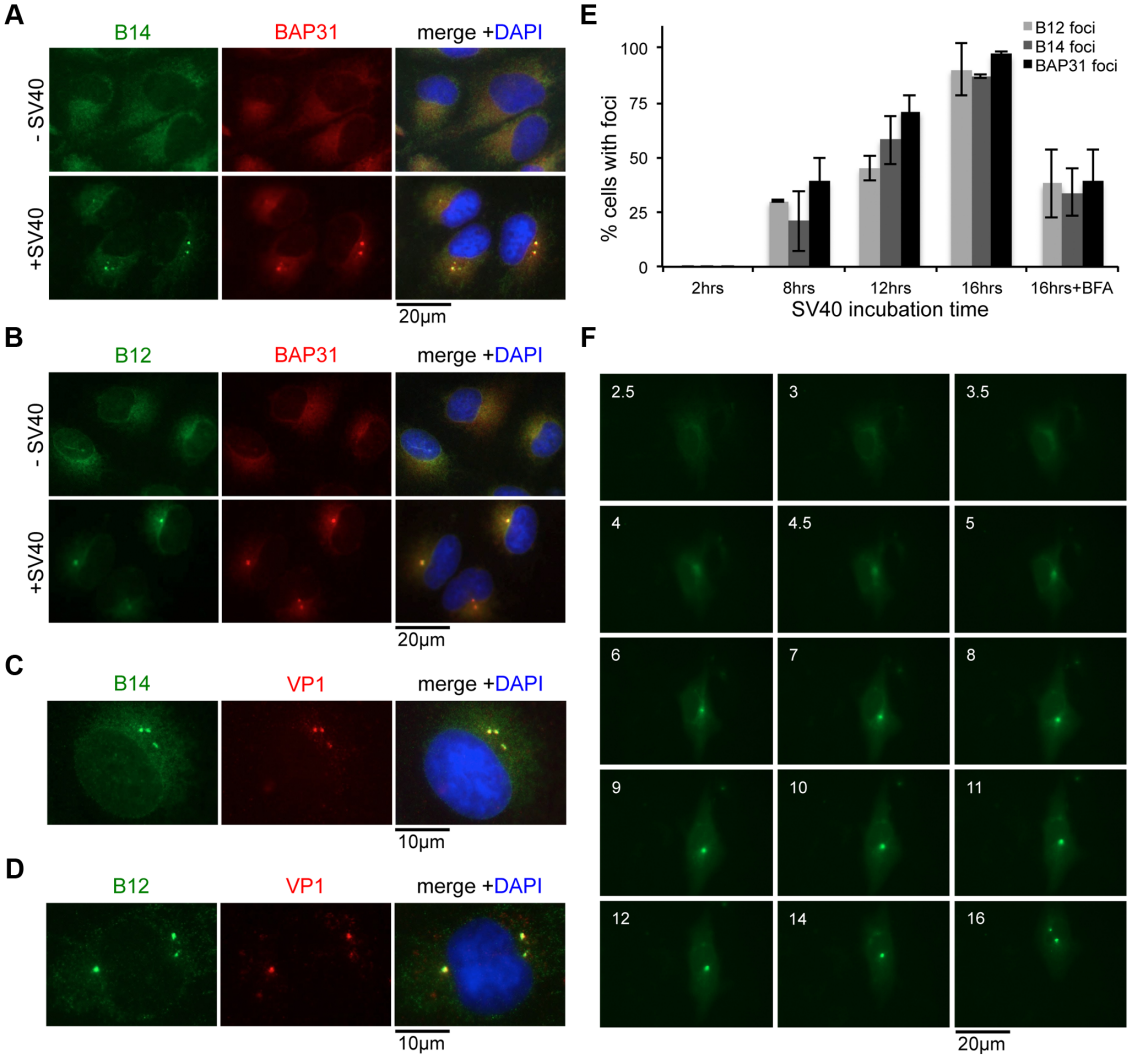
SV40 induces the B14-B12 complex to form foci on the ER membrane. (A) B14 reorganizes into foci upon SV40 infection. CV-1 cells uninfected or infected with SV40 (MOI ≈30–50) for 16 h were fixed and stained with the indicated antibodies for immunofluorescence microscopy. (B) B12 reorganizes into foci upon SV40 infection. As in (A) except B12 antibodies were used. (C) VP1 is present in B14-B12 foci. As in (A) using B14 and VP1 antibodies. (D) As in (C) except using B12 antibodies. (E) Time course experiment monitoring foci formation. Cells were infected as in (A). Where indicated, BFA was added during entry. Cells were fixed at different time points after infection and stained with antibodies against BAP31, B14, or B12. Cells were scored positive if at least one focus was present in the cell. Values represent mean ± SD of three independent experiments. (F) Live cell imaging of GFP-B14 foci formation. Cells were transfected to express B14 with an N-terminal GFP tag. Cells were infected with SV40 and live cell imaging performed beginning at 2.5 h.p.i. Numbers in each frame indicate time in h.p.i.

Our finding that SV40 liberates SGTA and Hsc70 from the B14-B12 complex suggests that the virus may restructure B14-B12 within the ER membrane. To assess whether SV40 imparts any reorganization of B14-B12, we stained endogenous proteins in fixed cells uninfected or infected with SV40 for analysis by immunofluorescence microscopy. In uninfected cells, both B14 and B12 colocalized extensively with the ER membrane protein BAP31 (Figure 6A and 6B, top panels), as expected. Strikingly, in infected cells, a significant portion of B14 and B12 was observed to concentrate into discrete foci within the ER (Figure 6A and 6B, lower panels). Some cells were observed to contain a single focus, while others contained several. Notably, B14 and B12 foci colocalized with VP1 (Figure 6C and 6D), suggesting that SV40 is responsible for foci formation. As reported previously, BAP31 also reorganizes into foci during SV40 infection [29]. We found that virus-induced BAP31 foci colocalized with the B14 and B12 foci (Figure 6A and 6B). These results indicate that, while B14-B12 and BAP31 were identified independently to be critical for SV40 membrane penetration, they are likely to be unified in facilitating this entry step rather than functioning in distinct parallel pathways.

The localization of Sec61α, a major translocon component reported to associate with BAP31 [56], remained diffuse and unchanged by addition of SV40 (Figure S2A, top panels). Similarly, Hrd1 staining revealed it did not form foci upon SV40 infection (Figure S2A, middle panels). Although Hrd1 is a central component of ERAD machinery [53], [57], it is dispensable for SV40 entry and infection [29]. Consistent with our observation that SGTA disengages from B14-B12 during entry, we did not detect any SV40-induced enrichment of SGTA in the B14-B12-BAP31 foci (Figure S2A, bottom panels). These data indicate that SV40 induces the reorganization of only a specific subset of ER membrane proteins into a discrete region.

We observed that knockdown of SGTA did not inhibit foci formation (Figure S2B), suggesting SGTA does not act prior to foci formation. We further characterized SV40-induced foci formation by performing time course experiments where the presence or absence of this structure was monitored during infection by staining with specific antibodies after fixation (Figure 6E). When cells were infected for 2 h, foci were not detected. After 8 and 12 h of SV40 infection, approximately 25–70% of cells contained B12, B14, and BAP31 foci. By 16 h, nearly every cell was positive for B12, B14, and BAP31 foci. BFA strongly inhibited foci formation. Moreover, we used live cell imaging to monitor foci formation in cells transiently transfected to express GFP-tagged B14 (GFP-B14). GFP-B14 was observed to gradually reorganize into clear foci at approximately 5–6 h after the addition of SV40 (Figure 6F). We conclude that SV40-induced reorganization of B14-B12 and other membrane components is dependent on ER arrival of the virus, occurs approximately 5–6 h.p.i., and is largely irreversible. The timing of SV40's cytosolic arrival [40] correlates strongly with our detection of foci in the ER membrane, therefore we postulate that these virus-induced structures may represent ER exit/cytosol entry sites for the virus.

### B14 Requires Its Lumenal Domain for SV40-Induced Foci Formation

**Figure 7:**
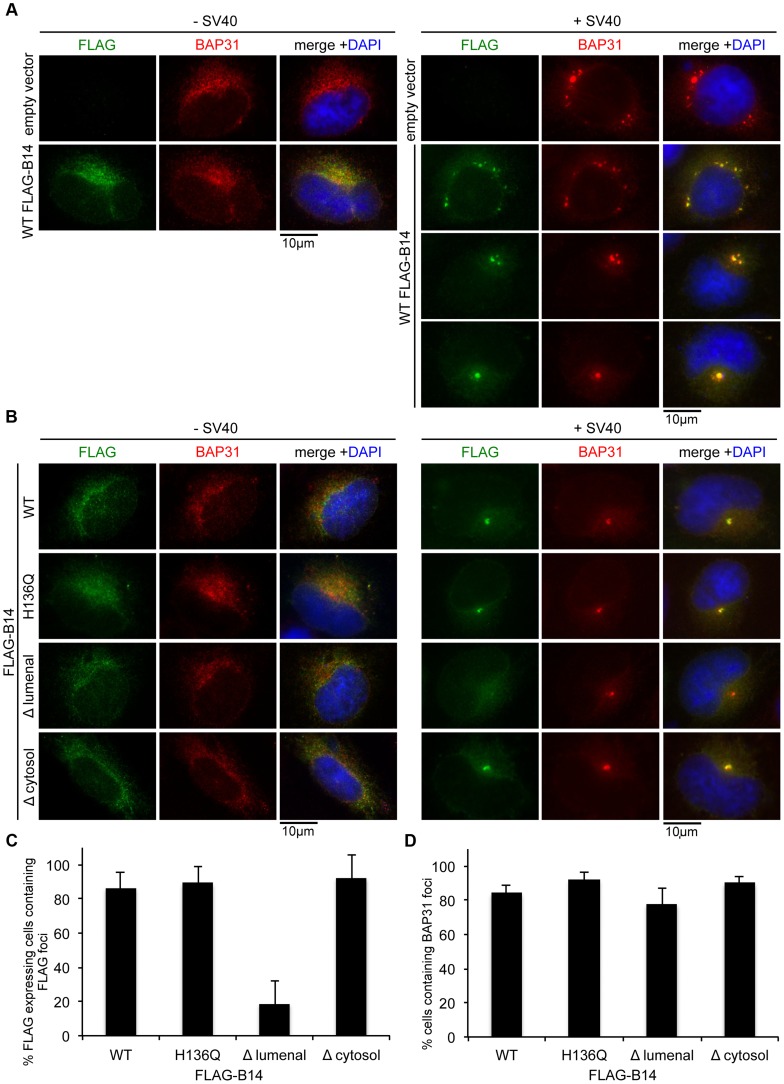
B14 requires its lumenal domain for SV40-induced foci formation. (A) Transfected WT FLAG-B14 forms SV40-induced foci. Cells were transfected with empty vector or a plasmid expressing WT FLAG-B14. Cells were uninfected or infected with SV40 for 16 h, fixed, and stained with antibodies for immunofluorescence microscopy. (B) Δlumenal FLAG-B14 fails to reorganize into foci. Cells were transfected with WT or mutant FLAG-B14 and analyzed as in (A). (C) Quantification of (B). Fixed cells were scored for the presence or absence of FLAG-foci in cells expressing the respective FLAG-tagged protein. Values represent mean ± SD of three independent experiments. A statistically significant (*p*<0.05) difference was observed with Δlumenal FLAG-B14 when compared to all other versions of FLAG-B14. (D) BAP31 foci formation is not disrupted by expression of WT or mutant B14. As in (C) except cells were scored for BAP31 foci in cells expressing the indicated FLAG-tagged protein. Values represent mean ± SD of three independent experiments. No statistical differences were found, *p*>0.05.

We sought to identify the molecular determinants within B14 necessary for SV40-induced B14 foci formation. When full length FLAG-tagged B14 was transfected into CV-1 cells and stained with FLAG antibodies, the transfected protein colocalized extensively with BAP31 in a diffuse manner (Figure 7A, left panels). When cells were infected, the ectopically expressed FLAG-B14 formed foci similar to the behavior of endogenous B14 (Figure 7A, right panels), with variable number of foci per cell observed. Comparable to endogenous B14 foci (Figure 6A), the ectopically expressed B14 foci also colocalized extensively with endogenous BAP31 foci.

As transfected WT B14 forms foci in response to SV40, we then asked whether transfected mutant forms of B14 are capable of forming foci during SV40 infection. Transfected H136Q B14, which does not interact with Hsc70 or SGTA, formed foci identical to WT B14 (Figure 7B, first and second row). Truncating most of B14's lumenal domain (97 amino acids) to generate Δ lumenal B14 strongly restricted its ability to form foci, despite this mutant colocalizing normally with BAP31 in the absence of SV40 (Figure 7B, third row; quantified in Figure 7C). In addition to the normal ER expression pattern, this mutant also precipitated endogenous B12 and SGTA (Figure S3), demonstrating the overall integrity of this variant. By contrast, a severe truncation of B14's cytosolic residues (i.e. Δ cytosol B14) did not restrict foci formation, acting similar to the full length WT B14 (Figure 7B, fourth row). Expression of the FLAG-B14 variants had minimal influence on the ability of endogenous BAP31 to form foci and did not perturb infection, likely due to the presence of endogenous B14 and sub-optimal transfection efficiency of CV-1 cells (Figure 7D). We conclude that the lumenal domain of B14 is required for its SV40-triggered reorganization into foci and potentially acts as a sensor for the virus directly.

### B14 Requires Its Lumenal Domain and Interaction with SGTA-Hsc70 to Promote SV40 Infection

**Figure 8:**
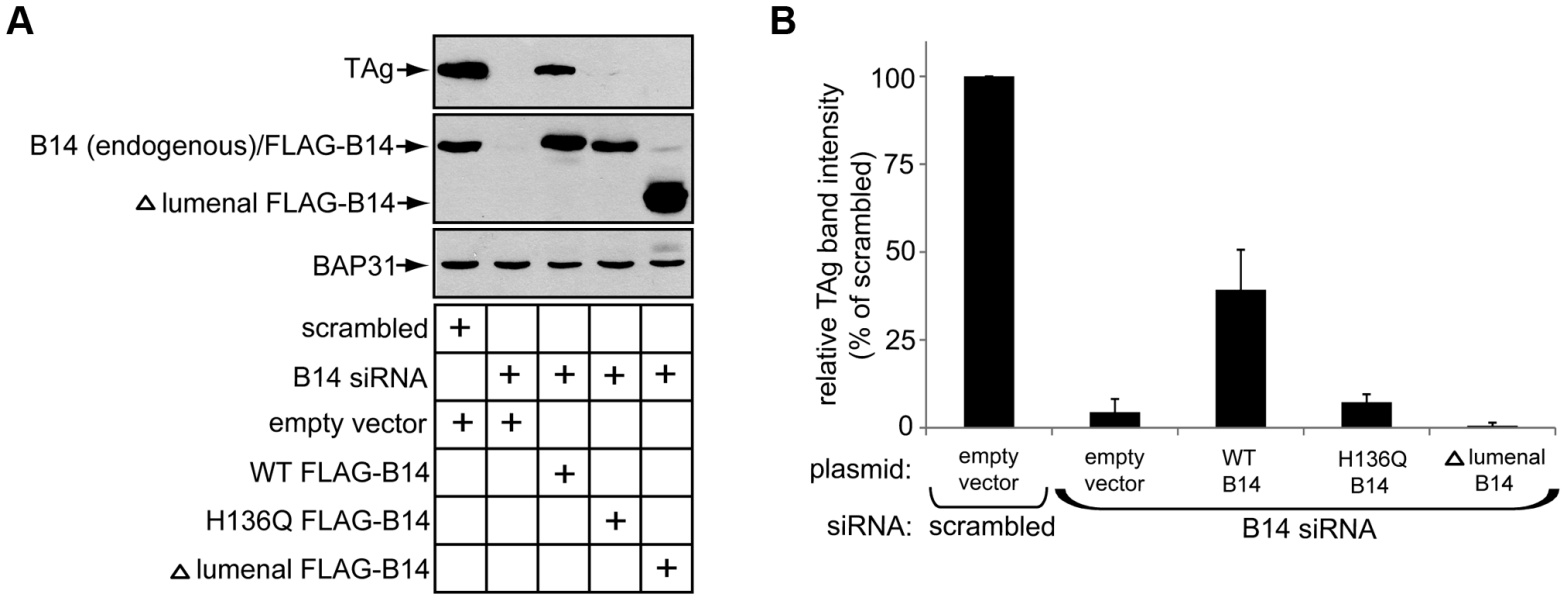
B14 requires its lumenal domain and SGTA-Hsc70 interaction to promote SV40 infection. (A) HeLa cells were transfected with the indicated DNA plasmids for approximately 18 h prior to transfection with scrambled or B14 siRNA for 48 h. SV40 was incubated with cells for approximately 40–48 h before whole cell lysates were prepared for immunoblot analysis with the indicated antibodies. (B) Quantification of (A), relative band intensities of TAg were determined using imageJ. Values represent the mean ± SD of three independent experiments.

We assessed the molecular requirements of B14 in promoting SV40 infection by performing rescue experiments. HeLa cells were chosen for these experiments because of their high transfection efficiency. DNA plasmids were first transfected for expression from an empty vector, WT B14, H136Q B14 or Δ lumenal B14. Subsequently siRNA transfections were carried out using a siRNA oligo targeting the 3′ UTR sequence of B14 to ensure depletion of only endogenous B14. After 48 h of siRNA treatment, cells were incubated with SV40 and successful infection measured 48 h later by immunoblotting for TAg. Infection of cells expressing empty vector as a control resulted in a complete loss of TAg expression upon B14 knockdown compared to scrambled siRNA (Figure 8A). This phenotype was reversed significantly in cells expressing WT B14. By contrast, H136Q B14 and Δ lumenal B14, while expressed at appropriate levels, failed to rescue infection in cells depleted of endogenous B14 (Figure 8A; quantified in 8B). These data support a model whereby B14's ability to engage SGTA-Hsc70 is necessary for SV40 infection. The inability of Δ lumenal B14 to form foci and promote infection suggests that foci may be functionally important for SV40 as well.

## Discussion

**Figure 9:**
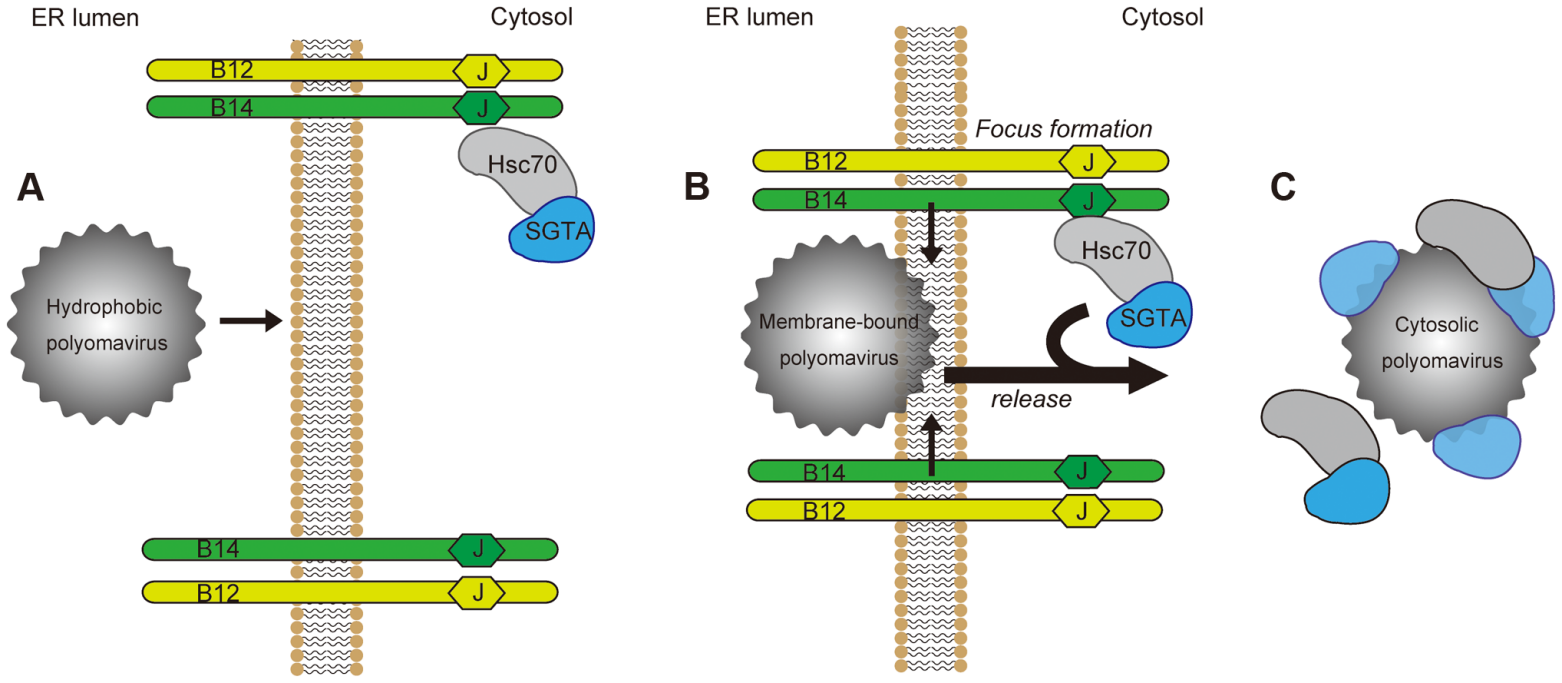
Model for ER-to-cytosol transport of polyomaviruses mediated by foci formation and cytosolic SGTA. (A) Polyomaviruses become hydrophobic due to conformational changes imposed upon them by ER lumenal chaperones and PDI proteins. This hydrophobicity allows for membrane binding and integration by the viral particle. (B) Membrane-bound viruses recruit the B14-B12 complex causing their reorganization into discrete foci, which may serve to transiently increase the local concentration of chaperones at the site of membrane penetration. (C) Polyomaviruses are mobilized out of the ER into the cytosol in a SGTA-dependent manner. SGTA and Hsc70 are released from the B14-B12 complex as SGTA physically interacts with the virus.

To cause infection, nonenveloped viruses must penetrate a biological membrane to gain access into the target cell. While host cues priming these viruses for membrane penetration are well-characterized, cytosolic factors co-opted to complete this membrane penetration event remain unknown. In this study, we pinpoint SGTA as a cytosolic chaperone that promotes membrane penetration of SV40 and likely other polyomaviruses. A model depicting SGTA-dependent SV40 ER membrane penetration is presented in Figure 9.

Our initial finding revealed that a pool of SGTA binds to the cytosolic surface of the ER membrane by engaging two transmembrane J-proteins called B14 and B12 (Figure 9A), essential factors for SV40 ER-to-cytosol transport and infection [33]. Interaction between SGTA and the B14-B12 complex requires Hsc70, consistent with previous reports demonstrating that SGTA interacts with Hsc70 [44]. The interaction of SGTA with the B14-B12 complex prompted us to ask whether it might serve to dislocate SV40 into the cytosol from the ER membrane. Indeed, our functional studies demonstrated that SGTA downregulation disrupts SV40 and BKPyV infection by blocking virus ER-to-cytosol transport, with subsequent binding experiments establishing a direct SGTA-SV40 physical interaction. These results suggest that SGTA engages the virus on the cytosolic surface of the ER membrane to mobilize it into the cytosol.

The use of SGTA to liberate SV40 into the cytosol from the ER membrane resolves a previous enigma in the mechanism of ER membrane penetration by SV40. Structural alterations occurring within the ER render the virus hydrophobic, enabling it to bind to and integrate into the ER membrane [27], [29]. Despite these remodeling events, SV40 remains a large and intact particle when it penetrates the ER membrane [40]. How this large and hydrophobic viral particle avoids aggregation in the aqueous cytosolic environment is unclear. Particle aggregation would clearly prevent SV40 from successfully reaching the nucleus to cause infection. One possibility entails cytosolic chaperones positioned at the ER membrane binding to and extracting the hydrophobic virus into the cytosol, and by use of this interaction, protecting the hydrophobic viral surfaces. Our results support this scenario by implicating SGTA, a cytosolic chaperone that binds to hydrophobic proteins [42], in mobilizing the virus into the cytosol and concomitantly protecting the viral hydrophobic surfaces.

Although SGTA has been recently linked to the ER-to-cytosol transport process known as ERAD [42], its apparent role in this instance is to assist the Bag6 complex in capturing ERAD substrates in the cytosol and preventing their aggregation prior to proteasomal degradation [42], [46]. By contrast, a separate study found that SGTA antagonizes Bag6 function during protein quality control in the cytosol [45]. Regardless of their relationship, SGTA appears to promote SV40 infection independently of Bag6. Our data reveals that SGTA likely serves additional undocumented roles in ER biology, possibly in cooperation with B14-B12.

Intriguingly, we observed that SGTA and Hsc70 disengage from the B14-B12 complex, an event that coincides with the drastic reorganization of the B14-B12 complex into foci on the ER membrane (Figure 9B). The energy source driving release of the virus-SGTA-Hsc70 complex into the cytosol is not known. It is possible that virus-induced B14-B12 foci formation imparts a conformational change that weakens the affinity between B14-B12 and SGTA-Hsc70. While SGTA itself does not harbor ATPase activity, it can modulate the ATPase activity of Hsc70 [43]. Thus, if SV40 binding to SGTA promotes SGTA to drive Hsc70 preferentially to the ADP-bound state, ADP-bound Hsc70 would have lower affinity for its cognate J-proteins B14 and B12 [58], [59]. This postulated scenario could explain how SV40 triggers release of SGTA-Hsc70 from B14-B12. Future experiments will clarify the precise mechanism by which SV40 induces SGTA and Hsc70 to disengage from the B14-B12 complex. Although the precise physiological significance of this observation is not entirely clear, discharge of the SGTA-Hsc70 complex bound with virus into the cytosol from the ER membrane (Figure 9C) is conceptually consistent with the requirement of SV40 to reach the cytosol in preparation for nuclear import. As Hsc70 proteins are observed to disassemble murine polyomavirus *in vitro* [55], polyomaviruses entering a host-cell could in principle co-opt both SGTA and Hsc70 activities to couple the cytosol release and viral disassembly reactions, with the latter likely being necessary for subsequent nuclear import.

The B14-B12 foci were positive for VP1, similar to a previous report that found VP1 colocalizes with BAP31 foci [29]. As ER membrane penetration is an inefficient process [40], only a small fraction of virus in the foci are expected to be released into the cytosol, explaining why VP1 accumulates in the foci at later time points. These foci unlikely represent nonspecific aggregation structures as no dramatic changes in the solubility of the B14 and B12 membrane proteins were observed during foci formation. Moreover, B14-B12 foci colocalize with BAP31, another ER membrane factor involved in SV40 ER membrane penetration [29], but not with other membrane proteins dispensable for SV40 infection such as a core ERAD component Hrd1. Thus foci consist of specific ER factors that conduct SV40 across the ER membrane but not ER components irrelevant to this process. Intriguingly, there is a temporal correlation between foci formation and virus arrival in the cytosol. Foci formation can be readily identified prior to 8 h.p.i. and SV40 cytosol arrival occurs between approximately 6–8 h.p.i. [29], [40]. Collectively, these observations raise the possibility that the B14-B12 foci may represent cytosol entry sites that function to increase the transient recruitment of SGTA-Hsc70 precisely at the membrane penetration site where SV40 can be efficiently extracted into the cytosol. It remains possible that smaller foci structures, which are not clearly detected by microscopy, also exist to mediate viral transport. This would explain why a stable SGTA-B14 interaction appears fully disrupted at time points where not all cells have visible foci.

A direct interaction between SV40 and B14-B12 or BAP31 has yet to be demonstrated, possibly due to a weak affinity of these factors to the particle within the membrane. This weak physical interaction could also explain the necessity of foci formation, which would facilitate multivalent interactions among several membrane components functioning to promote membrane penetration. At present, the relationship between B14-B12 and BAP31 remains obscure. Although these membrane proteins form foci in response to SV40 entry, no obvious physical interaction between B14-B12 and BAP31 can be isolated. Moreover, while BAP31 is thought to recognize membrane integrated SV40 with exposed VP2 via charge-pairing [29], both B14 and B12 lack charged residues within their transmembrane domains and thus recognize SV40 differently. In this regard, by evaluating different B14 mutants, we found that the lumenal portion of B14 is required for its SV40-induced reorganization and its ability to promote infection. This region of B14, which lacks any clear protein-protein interaction domains, could therefore act as a sensor for engaging SV40 complementarily with BAP31.

An outstanding question is whether a bona fide protein-conducting channel exists to accommodate SV40 transport across the ER membrane. If this is the case, it is unlikely that a native channel can support the transport of such a large viral particle whose diameter is approximately 45–50 nm [40]. Instead, the hydrophobic virus might initially integrate into the membrane and subsequently recruit and oligomerize ER membrane proteins such as B14, B12, and BAP31. The oligomerized structure (i.e. foci) would surround the viral particle as an intermediate until SGTA-Hsc70 extracts it into the cytosol.

In conclusion, this study identifies a novel cytosolic chaperone complex that completes ER membrane penetration of a nonenveloped virus, and utilizes dramatic rearrangement of ER membrane elements in the process. Viral entry, replication, and assembly are defining steps during the infection course. While diverse viruses are known to rearrange the ER membrane to facilitate viral replication and assembly [60], essentially nothing is known regarding how viruses reorganize the ER membrane during entry. The possibility that SV40 and other polyomavirus family members might reorganize components of the ER membrane to fashion its own entry site would demonstrate that viruses have the capacity to restructure the ER membrane to accommodate the early events of infection.

## Materials and Methods

### Antibodies

Polyclonal DnaJB14, DnaJB12, ERdj5, SGTA, Hrd1 were purchased from Proteintech Group (Chicago, IL). An additional rabbit polyclonal against SGTA was provided by Yihong Ye (NIH). Monoclonal BAP31 and polyclonal Hsc70 and Bag6 antibodies were purchased from Pierce (Rockford, IL). Polyclonal Derlin-1 and Sec61α antibodies were provided by Tom Rapoport (Harvard University). Normal rabbit and mouse IgG, polyclonal Hsp90, PDI, and monoclonal SV40 Large T antigen antibodies were purchased from Santa Cruz Biotechnology (Santa Cruz, CA). Rabbit anti-VP1 antibody was a gift from Harumi Kasamatsu (UCLA). Monoclonal VP1 antibody was provided by Walter Scott (University of Miami). Monoclonal p97 was purchased from RDI/Fitzgerald (Concord, MA). Polyclonal BiP and rat anti-Hsc70 antibodies were purchased from Abcam (Cambridge, MA). Polyclonal calnexin antibodies were purchased from Stressgen. Polyclonal ERp29 was a gift from Souren Mkrtchian (Karolinska Institutet).

### Reagents

Dulbecco's modified Eagle's medium (DMEM), Opti-MEM, 0.25% trypsin-EDTA were purchased from Invitrogen (Carlsbad, CA). Fetal Clone III (FC) was from HyClone (Logan, UT). Complete-mini EDTA-free protease inhibitor cocktail tablets were purchased from Roche. Micro Bio-Spin P-30 Tris chromatography columns were purchased from Bio-Rad. Dithiothreitol (DTT), Dithiobis succinimidylpropionate (DSP), N-ethylmaleimide (NEM) and anti-FLAG M2 agarose beads were purchased from Sigma (St Louis, MO).

### Preparation of SV40

WT SV40 and ΔVP2 SV40 were prepared using OptiPrep gradient system as described previously [40].

### Transfection of siRNA and DNA Plasmids

Control siRNA (labeled as scrambled) is the All Star Negative purchased from Qiagen (Valencia, CA). Custom siRNA sequences were generated and purchased from Dharmacon (Pittsburgh, PA) or Invitrogen.

B14 siRNA: 5′ GGUUCCUGAAAUCUUGGACUGUUUA 3′

5′ UAAACAGUCCAAGAUUUCAGGAACC 3′

SGTA siRNA #1: 5′ ACAAGAAGCGCCUGGCCUAUU 3′

5′ UAGGCCAGGCGCUUCUUGUUU 3′

SGTA siRNA #2: 5′ CAGCCUACAGCAAACUCGGCAACUA 3′

5′ UAGUUGCCGAGUUUGCUGUAGGCUG 3′

SGTA siRNA #3: 5′ CCAACCUCAAGAUAGCGGAGCUGAA 3′

5′ UUCAGCUCCGCUAUCUUGAGGUUGG 3′

Bag6 siRNA #1: 5′ GCUUGGAGGUGUUGGUGAAUU3′

5′ UUCACCAACACCUCCAAGCUU3′

Bag6 siRNA #2: 5′ GAUAAGAAGCUUCAGGAAUUU 3′

5′ AUUCCUGAAGCUUCUUAUCUU 3′

Using Lipofectamine RNAiMAX (Invitrogen), 25 nM (B14 or Bag6 siRNAs) or 12.5 nM (SGTA siRNAs) of control or custom siRNAs were reverse transfected into HeLa or CV-1 cells. Infection or biochemical assays were carried out 24 or 48 h.p.i.. A plasmid expressing human WT B14 was a gift from Daniel DaMaio (Yale) and cloned with an N-terminal FLAG tag into pcDNA3.1 minus (Invitrogen). Site-directed mutagenesis was performed on His 136 to yield a H136Q mutant. The Δlumenal FLAG-B14 contains residues 1-282 and Δcytosol contains residues 226-379, and were generated using standard cloning methods. A plasmid expressing SGTA-myc/FLAG is from Origene (Rockville, MD) and double point mutants were derived similarly by site-directed mutagenesis. DNA was transfected into plated CV-1 cells (50–80% confluent) using FuGENE HD (Promega) and allowed to express for 18–24 h prior to experimentation.

### B14-B12 and SGTA Co-immunoprecipitations

Interactions detected between endogenous B14 and B12 utilized lysates derived from CV-1 or HeLa cells with buffer containing 50 mM Tris pH 7.4, 150 mM NaCl, 1 mM EDTA and protease inhibitors with 1% deoxyBigCHAP (Calbiochem, Billerica, MA). Cleared lysates were incubated with antibodies overnight at 4°C with rotation. Antibodies were captured using protein A agarose beads and washed with lysis buffer containing 0.1% deoxyBigCHAP. SDS sample buffer was used for elution at 95°C. Interactions between endogenous SGTA and B14-B12 were detected similarly but with a different lysis buffer. 293T or CV-1 cells were lysed by resuspension and brief vortexing in 0.1% digitonin, 50 mM Tris, 150 mM NaCl, 1 mM EDTA and protease inhibitors followed by incubation on ice for 15 minutes. Cleared lysates were incubated with indicated antibodies, captured with protein A agarose beads and washed extensively with buffer lacking detergent. Immunoprecipitation of transfected SGTA-myc/FLAG was performed as above but with a buffer containing 0.2% digitionin and incubation with anti-FLAG M2 agarose beads.

### SGTA-SV40 Co-immunoprecipitations

In 6 cm plates, 3×10^5^ CV-1 cells were transfected to express FLAG-tagged GFP or SGTA proteins for at least 18 h. SV40 particles (15 μg) were added to cells after synchronizing the entry at 4°C. Approximately 8 h.p.i., cells were harvested and cross-linked using 2 mM DSP at room temperature for 30 mins. After quenching, cells were lysed in 1% Triton X-100, 50 mM Tris pH 7.5, 150 mM NaCl, 1 mM EDTA, 20 mM NEM and protease inhibitors. Cleared lysates were immunoprecipitated with anti-FLAG agarose beads overnight and washed 3 times with lysis buffer. Bound material was analyzed by immunoblotting using rabbit anti-VP1 antibody.

### XBP1 Splicing Assay

As previously described [25] using primers against human XBP1:

5′ CGCGGATCCGAATGTGAGGCCAGTGG 3′ and

5′ GGGGCTTGGTATATATGTGG 3′.

### Gel Filtration Chromatography

HeLa cells were lysed in 1% Triton, 30 mM Tris pH 8, 150 mM NaCl, and 4 mM MgCl_2_. Cleared lysates were separated using lysis buffer and a Bio-Sil SEC 250 column (Bio-Rad, Hercules, CA). Forty fractions of 0.5 mL were collected and fractions 10-22 were analyzed by immunoblotting.

### Preparation of Purified Proteins

For purification of FLAG-tagged GFP, WT and mutant SGTA, 293T cells were transfected to express proteins for 48 h. Cells were lysed in buffer containing 1% Triton X-100, 50 mM Tris, 150 mM NaCl, 1 mM EDTA and protease inhibitors. Cleared lysates were incubated with anti-FLAG agarose beads and bound proteins washed extensively with lysis buffer. Proteins were eluted with FLAG peptide overnight and concentrated using centrifugal filters that also removes residual FLAG peptides. Optiprep purified SV40 was treated with 3 mM DTT and 10 mM EGTA for 45 min at 37°C to mimic ER induced conformational changes. A spin column was used to exchange the buffer with PBS and to remove DTT and EGTA.

### *In Vitro* Binding Assays

Binding reactions were carried out in 50 μL PBS containing 250 ng of SV40 (pretreated with DTT and EGTA as described above) with or without 1 μg of purified protein. Reactions incubated for 1 h at 25°C followed by the addition of 0.25 mM DSP at 4°C for 30 min to stabilize transient interactions. After quenching with excess Tris, immunoprecipitation with anti-FLAG agarose beads was performed and bound material analyzed by immunoblotting.

### Immunofluorescence Microscopy for Foci Formation

CV-1 cells were grown on 12 mm coverslips in 6 or 24-well plate for 24 h. Cells were treated with SV40 for the indicated time and then washed in PBS followed by fixation with 1% formaldehyde at room temperature. Cells were permeabilized with 0.2% Triton X-100 and blocked with 5% milk and 0.2% Tween. Primary antibodies were incubated for 1 h at room temperature, followed by fluorescent conjugated secondary antibodies for 30 min at room temperature. Coverslips were mounted with ProLong Gold (Invitrogen). Images were taken using an inverted epifluorescence microscope (Nikon Eclipse TE2000-E) equipped with 60× and 100× 1.40 NA objective and a Photometrics CoolSnap HQ camera. For over-expression studies, cells were transfected with the desired plasmid with FuGene (Promega) at least 24 h prior to imaging. For live-cell imaging, cells are seeded on 35 mm glass bottom tissue culture dishes (Greiner Bio-one, Germany). Imaging of the cells was performed from 2 to 20 h.p.i. using the microscope and objective mentioned above. The entire set-up was controlled by MetaMorph software (Molecular devices) and ImageJ software (NIH) was used for image processing, analysis, and assembly.

### SV40 and BKPyV Infection Assay

CV-1 cells transfected with siRNA for 24 h were incubated with SV40 at 37°C (MOI ≈0.5 or 5). At 20–24 h.p.i., cells were harvested for analysis by immunoblot or fixed and stained using antibodies against SV40 TAg as described previously [40]. For each infection experiment, at least 500 cells were counted in each condition. Approximately 30–50% or 5–10% of cells were positive for TAg in the control conditions when challenged at MOI ≈5 or 0.5, respectively.

Purified BKPyV and pAb416 against BK large T antigen were provided by Michael Imperiale (University of Michigan). CV-1 cells transfected with siRNA for 24 h were infected at M.O.I. ≈0.5. Cells were harvested after 40 h.p.i., and immunoblot performed for analysis of TAg expression.

### ER-to-Cytosol Transport and ER Arrival Assays

Performed as in [40]. SV40 was added (M.O.I ≈5) to CV-1 cells grown in 6 cm plates (∼80% confluent). Where indicated, BFA (Epicenter, Madison, WI) was added to the media at 2.5 μg/mL. After fractionation, 40% of the supernatant fraction was compared alongside 10% of the pellet fraction for VP1 immunoblot analyses, as only a portion of SV40 reaches the cytosol. Fractionation markers were analyzed with equivalent amounts of supernatant and pellet. ImageJ software was used for quantification of VP1 band intensities. CTA1 transport was monitored in CV-1 cells as described in [40]. Cells were intoxicated with CT at 10 nM for 90 mins. ER arrival assays were also performed as described previously [40].

### Immunopurification of B14 Binding Partners

Flp-In T-REx 293 cells (Invitrogen) were transfected with a pcDNA5/FRT/TO plasmid expressing WT B14-3xFLAG along with pOG44 plasmid expressing a Flp recombinase. Selection was carried out with media containing hygromycin and blasticidin over several weeks. Expression of B14-3xFLAG to near endogenous levels was induced with 5 ng/mL of tetracycline provided to the media for 16 h. Three confluent 15 cm plates were collected in PBS and lysed for 30 mins on ice in 2.5 mL of buffer containing 0.1% digitonin, 50 mM Tris pH 7.4, 150 mM NaCl, 1 mM EDTA and protease inhibitor. Lysate was cleared with centrifugation at 20,000g for 15 min. Lysate divided in half was incubated for 2 h at 4°C with 30 μL of anti-FLAG M2 agarose beads that was pre-incubated with or without 3xFLAG peptide (100 μL, 0.25 mg/mL). Agarose beads were washed extensively with buffer lacking detergent. Bound proteins were eluted overnight at 4°C with 3xFLAG peptide (200 μL, 0.25 mg/mL). Three subsequent elutions were performed for 1 h each, pooled and concentrated using centrifugal filters (Amicon Ultra 0.5 mL 3K membrane). SDS sample buffer was added and heated for 30 min at 37°C followed by SDS-PAGE and silver staining or immunoblotting. Bands excised from a silver stained gel were analyzed by mass spectrometry at Taplin Biological Mass Spectrometry Facility (Harvard Medical School).

### B14 Rescue Experiments

In 6 cm plates, 4×10^5^ HeLa cells were transfected with pcDNA3.1 (empty vector) or B14 constructs for at least 18 h. Then 2×10^5^ cells were reverse transfected with either scrambled or B14 siRNA into wells of a 6 well plate. Cells were infected with SV40 48 h post-siRNA transfection and harvested for immunoblot after another 48 h.

### Statistics

Quantitative data is presented as the mean of at least three independent experiments with standard deviation. Paired two-tailed Student's t-tests were used to acquire *p-*values.

### UniProtKB/Swiss-Prot Identifiers

**VP1** P03087-1, **VP2/3** P03093-1/2, **B14** Q8TBM8, **B12** Q9NXW2, **Hsc70** Q96I56 **SGTA** O43765, **BAP31** P51572, **Bag6** P46379, **TAg** P03070 **Hrd1** Q86TM6,

**ERp29** P30040, **ERdj5** Q8IXB1, **Calnexin** P27824, **Ubl4a** P11441, **Trc35** Q7L5D6, **GEMIN5** Q8TEQ6, **GEMIN4** P57678, **KIF11** P52732, **DDX20** Q9UHI6,

**EIF3H** O15372, **SMN2** Q16637

## Supporting Information

Figure S1 Bag6 knockdown does not block SV40 infection, related to Figure 3. (A) CV-1 cells were transfected with the indicated siRNAs for 24 h before being infected with SV40 for an additional 24 h and harvested. Immunoblot analysis was performed with the indicated antibodies.
(TIF)

Figure S2 Additional characterization of SV40-induced foci formation, related to Figure 6. (A) CV-1 cells were infected with SV40 for 16 h before being fixed and stained with the indicated antibodies for immunofluorescence microscopy. (B) CV-1 cells were transfected with scrambled or SGTA siRNA prior to addition of SV40. 16 h.p.i., cells were fixed and stained for BAP31. Cells were scored for the presence or absence of foci. Data are normalized to the scrambled condition and represent the mean ± SD of three independent experiments. No statistical differences were found, *p*>0.05.
(TIF)

Figure S3 B14 mutant lacking lumenal domain interacts with B12 and SGTA, related to Figure 7. (A) CV-1 cells were transfected to express FLAG-tagged B14 mutant or left untransfected. Lysates were prepared and immunoprecipitation carried out using anti-FLAG agarose beads. Bound material was analyzed by immunoblotting.
(TIF)
