# Supplementary figures and images for: A Cytosolic Chaperone Complexes with Dynamic Membrane J-Proteins and Mobilizes a Nonenveloped Virus out of the Endoplasmic Reticulum
Source: PLoS Pathog. 2014 Mar 27;10(3):e1004007. doi: 10.1371/journal.ppat.1004007 (PMC3968126; doi:10.1371/journal.ppat.1004007)

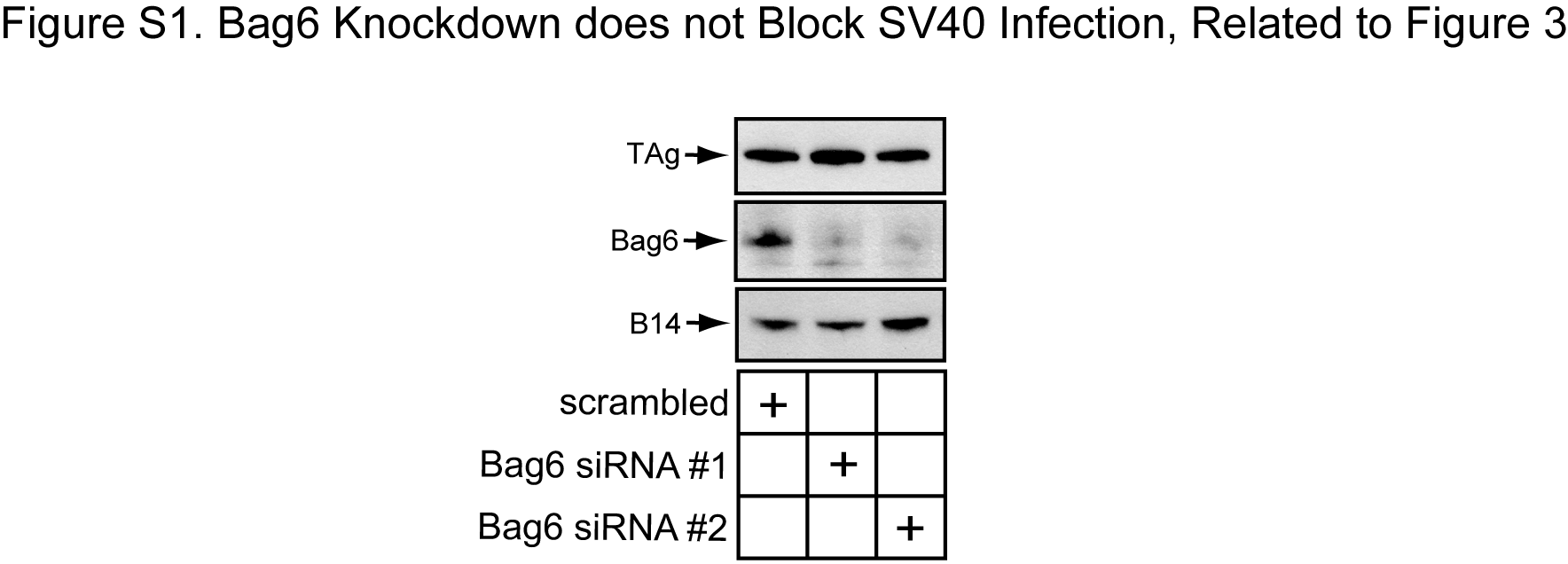

Supplement: Figure S1 — Bag6 knockdown does not block SV40 infection, related to Figure 3. (A) CV-1 cells were transfected with the indicated siRNAs for 24 h before being infected with SV40 for an additional 24 h and harvested. Immunoblot analysis was performed with the indicated antibodies. (TIF) [file ppat.1004007.s001.tif]

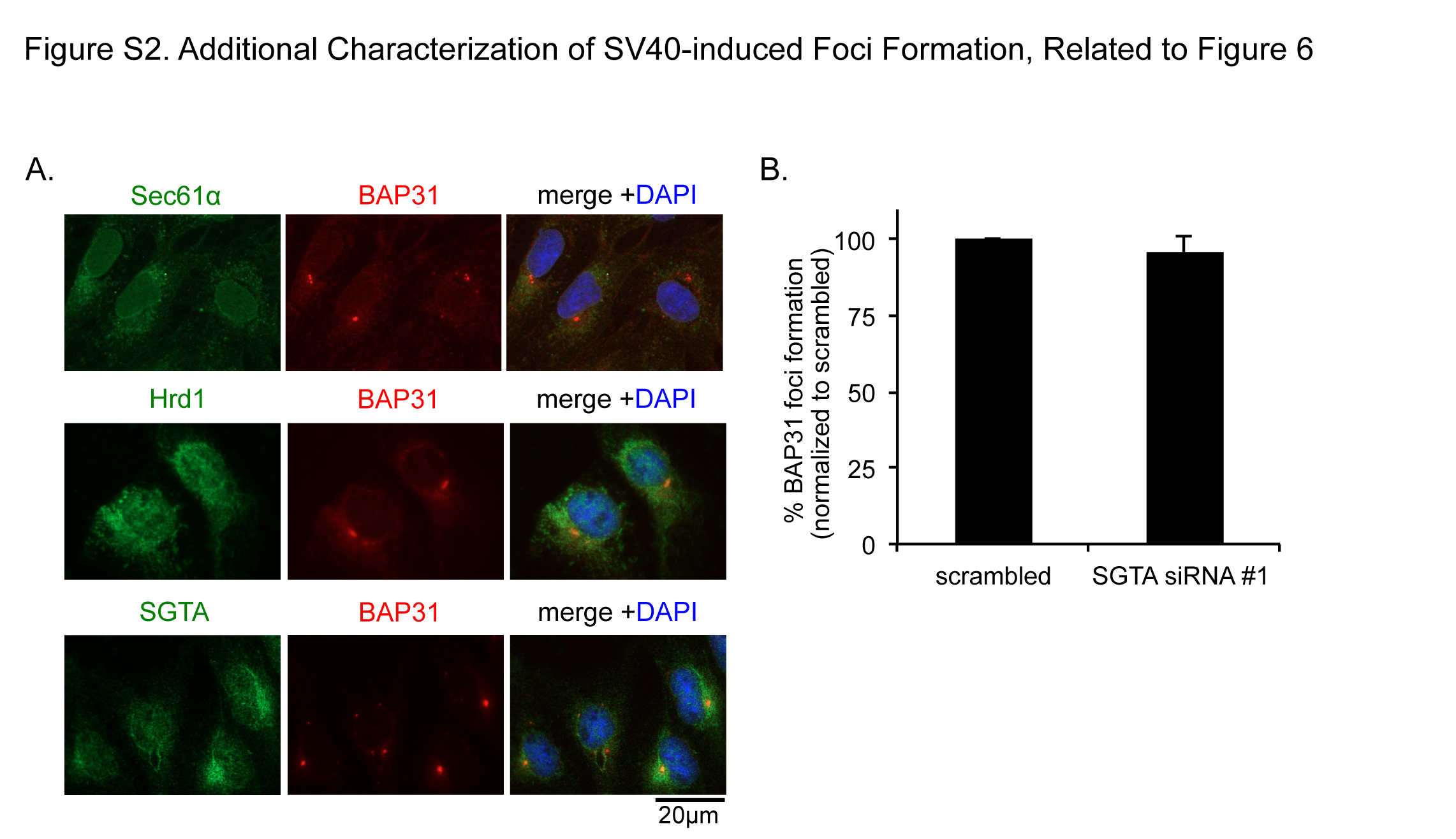

Supplement: Figure S2 — Additional characterization of SV40-induced foci formation, related to Figure 6. (A) CV-1 cells were infected with SV40 for 16 h before being fixed and stained with the indicated antibodies for immunofluorescence microscopy. (B) CV-1 cells were transfected with scrambled or SGTA siRNA prior to addition of SV40. 16 h.p.i., cells were fixed and stained for BAP31. Cells were scored for the presence or absence of foci. Data are normalized to the scrambled condition and represent the mean ± SD of three independent experiments. No statistical differences were found, p>0.05. (TIF) [file ppat.1004007.s002.tif]

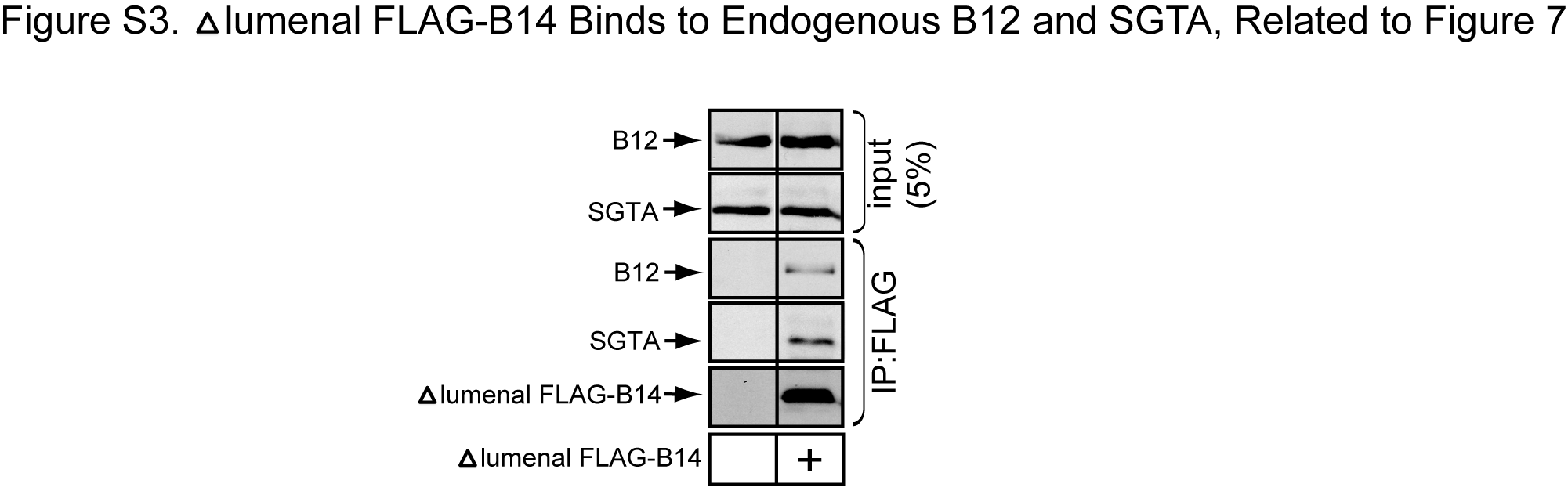

Supplement: Figure S3 — B14 mutant lacking lumenal domain interacts with B12 and SGTA, related to Figure 7. (A) CV-1 cells were transfected to express FLAG-tagged B14 mutant or left untransfected. Lysates were prepared and immunoprecipitation carried out using anti-FLAG agarose beads. Bound material was analyzed by immunoblotting. (TIF) [file ppat.1004007.s003.tif]
